# Nutrition-Based Strategies to Reduce Exercise-Induced Muscle Damage and Soreness

**DOI:** 10.3390/nu15112523

**Published:** 2023-05-29

**Authors:** Matthew J. Barnes

**Affiliations:** School of Sport, Exercise & Nutrition, Massey University, Palmerston North 4472, New Zealand; m.barnes@massey.ac.nz; Tel.: +64-6951-6822

Exercise induced-muscle damage (EIMD) occurs after strenuous and/or novel exercise that involves repeated eccentric contractions. A well-studied phenomenon, EIMD is associated with transient symptoms that include delayed onset of muscle soreness (DOMS), elevations of circulating biomarkers, such as creatine kinase (CK), myoglobin and lactate dehydrogenase, inflammation, and, perhaps most importantly, a decrease in the muscles’ ability to generate force. DOMS and force loss, in particular, can negatively impact an individual’s ability to exercise and may influence exercise adherence. In order to reduce the symptoms of EIMD, a number of strategies have been investigated including the use of various therapeutic modalities, including nutritional interventions [1] aimed at minimising EIMD and expediting recovery after exercise.

The potential for nutritional compounds, either in isolation or in whole food, is vast; therefore, the aim of this Special Issue was to advance our understanding of how nutrition can influence the responses to and outcomes of EIMD. This Special Issue includes six original articles investigating the effects of various foods on symptoms of EIMD. Additionally, Crum et al. [1] have reviewed many of the common compounds that have been previously investigated and, although many show promise, the majority currently lack the scientific evidence to fully support their use in optimising recovery. Of the compounds reviewed, tart-cherry and omega-3 fatty acids have the most compelling evidence for their use, while curcumin, pomegranate, creatine monohydrate, β-hydroxy β-methylbutyrate (HMB) and branch chain amino acids (BCAA) have a moderate level of evidence and may be worthy of consideration. However, Khemtong et al. [2] question the efficacy of using BCAAs and report that EIMD-related changes in perceived muscle soreness and neuromuscular performance were not improved by BCAA supplementation after repeated change of direction sprinting.

Protein, from animal sources, may aid in the repair of damaged muscle tissue; however, with the increased popularity of plant-based proteins, a better understanding of how plant-based protein compares to animal-based protein is needed. Supplementing the diets of older adults with either pea protein, whey protein or an isocaloric placebo for 13 days, Spoelder et al. [3] found that only whey protein reduced CK levels in the days after a bout of prolonged (~5 h) walking. Further supporting the potential benefits of dairy protein, Fraschetti et al. [4] illustrate the ability of cow’s milk to alter the post-eccentric exercise inflammatory response by reducing the relative inflammatory responses for IL-1β and IL-10.

New applications of common foods and traditional medicines add to our understanding and nutritional toolbox for dealing with EIMD. Two such examples are the findings of Lee et al. [5] and Yeh et al. [6] who report positive effects of *Lactobacillus paracei* and astragaloside supplementation, respectively, on muscle performance, biomarkers of muscle damage and inflammation. The common practice of daily consumption of probiotics to enhance gut health also appears to provide protection and enhance recovery from strenuous damaging exercise [5]. Sourced from the plant *Astragalus*, and used in many Asian countries as a herbal remedy, astragalosides have known antioxidant and anti-inflammatory properties, in addition to promoting myogenesis; these actions may be responsible for the recovery benefits reported by Yeh et al. [6].

Using computational systems biology analysis, Ayyadurai et al. [7] suggest a novel approach to reducing muscle soreness and maintaining muscle health. This study found that D-Glucaric acid reduces liver toxicity which, due to the fact liver detoxification has been shown to reduce muscle damage and support muscle recovery, may benefit recovery from EIMD. Using the framework established in this study, future research is needed to test this hypothesis.

In summary, the articles submitted to this Special Issue contribute to our understanding of how nutritional strategies can alter the responses to EIMD and highlight the diverse range of compounds and foods that may be beneficial for enhancing recovery from strenuous, damaging exercise.
